# Draft Genome Sequence of *Pseudomonas stutzeri* Strain B1SMN1, a Nitrogen-Fixing and Naphthalene-Degrading Strain Isolated from Wastewater

**DOI:** 10.1128/genomeA.00584-13

**Published:** 2013-08-08

**Authors:** Antonio Busquets, Arantxa Peña, Margarita Gomila, Joan Mayol, Rafael Bosch, Balbina Nogales, Elena García-Valdés, Antonio Bennasar, Jorge Lalucat

**Affiliations:** Microbiologia, Departament de Biologia, Universitat de les Illes Balears, Palma de Mallorca, Spaina; Institut Mediterrani d’Estudis Avançats (IMEDEA, CSIC-UIB), Palma de Mallorca, Spainb; Instituto Universitario de Investigaciones en Ciencias de la Salud (IUNICS-UIB), Universitat de les Illes Balears, Campus UIB, Palma de Mallorca, Spainc

## Abstract

*Pseudomonas stutzeri* strain B1SMN1 is a naphthalene-degrading and simultaneously nitrogen-fixing strain isolated from a wastewater sample taken at a lagooning treatment plant in Menorca (Balearic Islands, Spain). Here we report the draft genome sequence of *P. stutzeri* B1SMN1. It is composed of a chromosome of an estimated size of 5.2 Mb and two plasmids of 44,324 bp and 56,118 bp.

## GENOME ANNOUNCEMENT

*Pseudomonas stutzeri* strain B1SMN1 was isolated in 1988 from a sample taken at a wastewater treatment plant in Menorca (Balearic Islands, Spain) in mineral medium with 2-methyl-naphthalene as the carbon and energy source (1). This strain was classified as a member of genomovar 1 (gv1) of *Pseudomonas stutzeri* (2, 3). In a screening of *P. stutzeri* strains able to fix dinitrogen, the B1SMN1 strain was selected for its ability to degrade naphthalene and simultaneously fix dinitrogen under microaerophilic conditions, which is a relevant property in the biodegradation of pollutants in environmental biotechnology. The chromosome size of strain B1SMN1 was estimated by macrorestriction fragment analysis, and the presence of two circular plasmids not harboring naphthalene-degrading genes was also demonstrated (4).

The draft genome sequence of *P. stutzeri* strain B1SMN1 was *de novo* assembled with Newbler Assembler version 2.7 (Roche), combining 200,929 reads from 454 Titanium (mean read length 500 bp) with 657,175 reads from Ion Torrent (mean length 200 bp). The obtained genome sequence included 74 contigs (>500 bp). The estimated genome size is 5.2 Mb, the highest so far described for a *P. stutzeri* strain. Additionally, two plasmids of 44,324 bp (contig 29) and 56,118 bp (contig 20) were found. The G+C mole percent values are 65.32% for the chromosome and 61.4% and 63.44% for plasmids.

The genome prediction and annotation was performed using the NCBI Prokaryotic Genomes Automatic Annotation Pipeline (http://www.ncbi.nlm.nih.gov/genomes/static/Pipeline.html). A total of 4,931 coding sequences were identified in the chromosome. Genes coding for discriminating metabolic and physiological properties of the species were detected, including the complete set of genes for the denitrification pathway, for starch metabolism, and for flagellum synthesis. In the chromosome, a complete set of nitrogen-fixation genes was found (contig 7; 37.3 kb), as well as the upper and lower operons for naphthalene and salycilate degradation, which were distributed in three contigs. Predicted phage-related sequences, transposons, integrons, and insertion elements were detected. Of the 60 encoded proteins in plasmid 1, 32 were annotated as hypothetical proteins, 16 were related to conjugation (9 of them were related to type IV secretion factors), and 12 were related to plasmid biology. In plasmid 2, 64 encoded proteins were found; 20 were annotated as hypothetical proteins and 6 as transposases, and 38 were related to plasmid biology and conjugative events.

Whole-genome sequences of 13 *P. stutzeri* strains are publicly available (5–12). Four of them are also considered nitrogen fixers. Comparative genome analysis and GC content confirmed that strain B1SMN1 exhibited overall similarity to the *P. stutzeri* strains of the previously sequenced genomovars 1, 2, 3, 8, and 19. Average nucleotide identity calculated with the BLAST algorithm (ANIb) values (13), which discriminated the genomovars of the species, confirmed the adscription of strain B1SMN1 to gv1; values higher than 96% were found for strains of the same genomovar, 80 to 93% for strains of different genomovars, and lower than 77% with other *Pseudomonas* species.

### Nucleotide sequence accession numbers.

This whole-genome shotgun project has been deposited at DDBJ/EMBL/GenBank under the accession number AMVM00000000. The version described in this paper is the first version, AMVM10000000.
